# Benefits of workplace collaboration with a trauma-informed support service: a qualitative study

**DOI:** 10.1186/s12913-025-13398-x

**Published:** 2025-10-01

**Authors:** Jacinta Evans, Katherine Piech, Samantha Stark

**Affiliations:** Australian Health Practitioner Regulation Agency, Melbourne, Australia

**Keywords:** Trauma-informed, Implementation, Health practitioner regulation, Collaboration, Staff

## Abstract

**Background:**

Trauma-informed services can benefit people involved in a healthcare or legal process by supporting them to engage with systems and minimise risk of retraumatisation. Research also suggests that the integration of trauma-informed services can benefit staff members who work with these services. This study sought to explore outcomes for staff members following the introduction of a trauma-informed navigation and support service for victim-survivors involved in sexual boundary violation complaints lodged with the Australian health practitioner regulator.

**Methods:**

Researchers conducted a series of semi-structured interviews (*n* = 13) with regulatory staff members who had engaged with the support service. Interview transcripts were analysed using reflexive thematic analysis to qualify participant experiences and detail outcomes of working with the service.

**Results:**

Four main themes related to outcomes of collaborating with the support service were identified: (1) additional support for engagement work, (2) changes to interactions with victim-survivors (notifiers), (3) personal benefits of collaboration, and (4) improved perception of workload. These themes showed that staff identified positive collaboration with the service and benefits resulting from that collaboration. However, a negative case analysis identified perspectives that did not fit with the main interpretation of the data, where participants did not see a clear need for the service. This demonstrates some of the challenges implicit to integrating a new service within an organisation, including how perception of benefits can be limited due to conflicting personal or professional ideologies.

**Conclusions:**

Staff within a national regulator identified personal and professional benefits resulting from workplace collaboration with a trauma-informed service introduced to support victim-survivors. With the introduction of any new service, challenges can arise, and thoughtful planning is required prior and during implementation to understand individual and environmental factors that may impact integration.

**Supplementary Information:**

The online version contains supplementary material available at 10.1186/s12913-025-13398-x.

## Background

Experiencing trauma can have immediate and long-term adverse impacts on a person’s health and wellbeing [1]. Trauma can influence that person’s likelihood of seeking support or redress from healthcare systems and affect their level of engagement throughout the process [1].

Interaction with these systems often increases the risk of retraumatisation [2, 3]. In response, organisations around the world have moved towards implementing trauma-informed approaches [3]. A trauma-informed approach emphasises awareness, recognition and response to the effects of trauma [4] to safely navigate people through a process.

There are several methods of incorporating trauma-informed approaches, including an integrated care or ‘joined up’ model, where multiple services or professionals come together to provide wraparound support to service users [9]. These models often involve social workers, who are trained to deliver trauma-informed care that incorporates core principles of safety, trust, collaboration, choice, and empowerment [4]. Under models of integrated health care, social workers operate alongside health professionals to provide holistic support that can improve patient outcomes [5–7], particularly patients’ mental health [8]. Similarly, collaboration between social work and legal professionals can benefit people undergoing legal processes [9, 10].

Trauma-informed and integrated models can also add value for the professionals involved. By addressing social determinants of health, coordinating care across service providers, and improving communication with patients and families through these models [8, 11–13], social workers enable healthcare practitioners to focus on providing quality medical care. Some studies show that social workers in integrated settings provide practical and emotional support to practitioners that helps them manage the challenges of their work [14], and that attitudes towards social workers in integrated teams are generally positive [15]. More broadly, implementing trauma-informed models in the workplace can increase staff satisfaction [16] and positively influence working environments [17].

In 2021, the Australian Health Practitioner Regulation Agency (Ahpra) introduced an internal support service for victim-survivors^1^ involved in cases of an alleged sexual boundary violation by a health practitioner. A sexual boundary violation in this context can include allegations of sexualised comments, unwarranted intimate examinations, intimate examinations undertaken without appropriate informed consent, sexual relationships between practitioners and patients, sexual exploitation, harassment and assault. The service is led and delivered by social workers, who work closely with regulatory and legal staff to offer trauma-informed navigation and support through the regulatory process to victim-survivors while the case is being managed. This may involve clarifying process steps, liaising with legal or regulatory staff, or supporting victim-survivors to access external services. In introducing the service, Ahpra responded to feedback from internal stakeholders that identified a need to support victim-survivors to engage in the regulatory process and provide additional support to the staff members managing these cases.

While trauma-informed navigation and process support services have been successful in other settings [18, 19], the creation of a service to provide support during a health regulatory process was a novel and untested approach. As such, it was unclear whether staff would identify the kinds of positive outcomes demonstrated in other integrated collaborations more broadly. With this as a premise, the aim of this study was to explore staff perspectives of the support service following its introduction, with a focus on outcomes for staff resulting from working with the service. The research question for this study was, “What were staff members’ experiences of collaboration with a trauma-informed service?”

## Methods

### Theoretical framework

A reflexive thematic analysis approach following Braun and Clarke’s (2021) process [21] was chosen to guide analysis of interviews. This approach centres the “researcher’s reflective and thoughtful engagement with their data and their reflexive and thoughtful engagement with the analytic process” [23] and aims to reach richer interpretations of meaning through collaboration rather than attempting to achieve consensus [23].

### Setting

The setting for this study is a national health practitioner regulator in Australia. The regulator employs more than 1,000 people [20] in diverse roles across the country, with a shared overarching aim of protecting the public. When a member of the public, health practitioner, or employer has concerns about the safety of a health practitioner’s performance, health, or conduct, they can raise a concern (termed a notification) with the regulator, who will then assess and if necessary, investigate the concern. Ahpra may then work with decision makers to consider regulatory action. This work is the responsibility of staff in regulatory and legal services functions (hereafter referred to as ‘staff’ regardless of function, unless specified). Staff in both functions can refer victim-survivors (described as ‘notifiers’ and/or ‘witnesses’ by staff members) to the support service.

### Recruitment and sampling

Participants were recruited using a purposive sampling strategy to identify staff members who had referred to and engaged with the support service. As the project was exploratory and sought to capture a wide range of perspectives, all staff who had referred participants to the service in the three months prior to data collection were identified via referral data and cross-checked with social workers’ activity records to determine case engagement. Engagement was agreed to mean that the referring staff member had multiple instances of activity, such as email, phone, or videocall communication, recorded by the social workers in at least one matter following that referral.

Staff identified as having referred to and engaged with the service within the study timeframe were contacted by a member of the research team via email to obtain consent to contact, after which study information including a detailed participant information sheet was sent to them for review.

### Data collection and analysis

Interviews were organised with individual participants via email and were conducted and audio recorded via video call with JE, KP, or both (*n* = 1) using Microsoft Teams for Windows (Microsoft, 2023) or Zoom for Windows (Zoom Communications, 2023) according to participant preference. Prior to interviews commencing, researchers confirmed that their roles were wholly separate to regulatory or legal functions, outlined the details, purpose, and proposed outputs of the study, reiterated anonymity of data for transcription, and reminded participants they could withdraw at any time without giving a reason. Verbal informed consent to participate was provided by participants verbally in interviews, which then proceeded following a semi-structured question schedule developed for this study (Additional file 1). Researchers closed the interviews by confirming contact details for any questions or concerns. Follow up interviews were not conducted.

Interview audio recordings were sent to a confidential third-party service for transcription. Deidentified transcripts were imported to NVivo 14 (Lumivero, 2023) for analysis following Braun and Clarke’s (2021) reflexive thematic analysis process [21]. Researchers (KP and JE) first completed several close readings of all transcripts before beginning to code data separately based on patterns and key points identified in each transcript. Researchers met regularly to discuss the development of codes, creating a combined structure and revisited each transcript to re-code to that structure. Themes were developed over time and through frequent, in-depth engagement with data facilitated by discussions within the research team.

To help guide the analysis, researchers adopted an inductive thematic approach to data saturation [22]. Some scholars dispute the utility of reporting saturation [23], especially as a single indicator of validity [22]. Rather than a distinct point at which saturation was ‘achieved’, sufficient saturation was agreed between researchers during analysis when repeated engagement with the data did not lead to additional codes, suggesting that the coding structure was representative of the content and appropriately met the aims of this project. This was consistent with inductive analysis and the inductive thematic approach to saturation.

### Reflexivity and positionality

Reflexive qualitative research requires that the backgrounds and characteristics of the researchers be considered in reference to findings, and further, that researchers engage with their subjectivity rather than disregard or deny it [24]. All research team members are female, have relevant qualitative research experience, and have been involved in related work as part of their roles in Ahpra’s Research, Evaluation and Insights team [25].

Researchers used reflexivity tools including checklists, working journals, and debriefing throughout the course of the project to critically interact with subjectivity and support nuanced analysis of the findings.

Researchers were careful to consider their position in relation to regulatory and legal staff, as well as their working relationship with staff of the support service during all stages of the work. The researchers who conducted interviews did not have pre-existing relationships with staff members who were invited to participate. Interviewers clearly iterated before interviews began that their roles were distinct from regulatory operations. Participants were invited to opt-in to receive updates on dissemination of the work.

Recruitment data, interview recordings and transcripts, and all notes and records of meetings were all stored on a secure server not accessible to staff beyond the research team.

## Results

### Participants

Twenty-nine current staff members were identified as having referred participants and engaged with the service at the time of data collection. Of these, 17 agreed to participate in an interview. Four staff members could not be interviewed due to scheduling conflicts, leave, or staff exiting the organisation during the data collection period. Thirteen participants were interviewed and formed the sample for the study. Five participants were employed within the Notifications function and eight were employed within Legal Services or Legal Support. Participants had varied length of employment at Ahpra and degree of experience with the service. For confidentiality purposes, potentially identifiable demographic data is not reported here, and participants are identified using number codes in the findings presented below.

### Interview findings

**Fig. 1:**
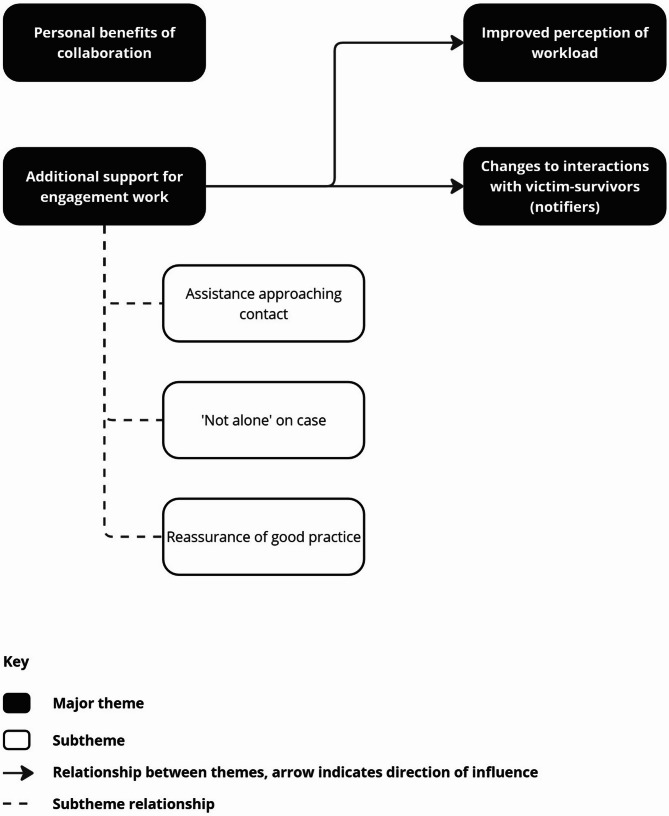
Themes and relationship

The median interview duration was 29 min, and interviews ranged from 17 to 43 min in length. In interviews, participants reflected on their experiences since the introduction of the service and reported a range of outcomes that came from engaging with the service. These are collated in Additional file 2, and are presented under four major themes: (1) Additional support for engagement work, (2) Changes to interactions with victim-survivors (notifiers), (3) Personal benefits of collaboration, and (4) Improved perception of workload. Figure 1, below, summarises themes, subthemes, and relationships.

Through additional support for engagement work, which included assistance approaching contacts, feeling ‘not alone’ on cases, and being reassured of good practice in their communication, participants reported a perceived reduction of their workload as well as changes to their interactions with victim-survivors. The collaboration more generally brought personal benefits including positive working relationships and reduced stress in some cases.

During the course of analysis, a negative case was identified that did not fit within this central interpretation of the data. The case is also reported here to ensure that this variation is contextualised and accounted for in the study’s conclusions.

#### Theme 1: additional support for engagement work

Participants described feeling that they had additional support to engage with victim-survivors when they collaborated with the service. Three subthemes were evident within this main theme, covering reports of assistance in planning communication, feeling ‘not alone’ on cases, and receiving reassurance of good practice.

Participants spoke of social workers assisting staff before, during, and after contact with victim-survivors, including preparing for phone calls and meetings, and providing support drafting correspondence.

*“We all talk with the [service] to form a strategy about how we’re going and what we’re going to be saying before we have conversations as well. So that’s just reassuring. I mean obviously I would do that before I’d call people if I had a tricky conversation coming up*,* but it’s much easier if you can have that plan [with someone] who has expertise in the area. It makes my job much less stressful.” (04)*.

This support helped staff feel less alone on cases with one participant describing this collaboration as “two people who are deal[ing] with a difficult situation together” (01). Importantly, the social workers and regulatory staff had equal understanding of the person at the centre of the process:

*“I wasn’t alone. We both had context of the person and their manner*,* personality*,* concerns*,* distress*,* expectations. As opposed to a case without it*,* we have – I have numerous*,* and have had numerous challenging cases with challenging practitioners or notifiers. And I guess when you’re seeking support of a team leader or a manager*,* they’re not in the conversation with you. It’s a little bit different. So*,* I guess it’s just – just from that physical connection. [Managers] are not hearing and listening to the context of a person. They’re hearing*,* you know*,* afterwards.”* [13].

Working with the service provided reassurance to staff engaging with victim-survivors that their communication would be better suited to the recipient.

*“Liaising with the [service] about how best to approach a patient who we think might not necessarily be particularly receptive to communication with us and just being able to sort of brainstorm how we’re going to approach that*,* and to have their insight because obviously I’m a lawyer and social work - that kind of side we’re not trained in*,* so it’s really helpful to be able to discuss that.”* [12].

*“I’ve often*,* you know*,* with [social worker] done like a bit of a sense check of*,* ‘oh do you think this will come across as okay?’*,* because I need to move along*,* for example*,* in an investigation*,* I need to give some bad news or something like that*,* and it’s really nice to have a social worker there to have that sense check with of ‘how do you think this will come across*,* do you have any ideas of how we might phrase this a little better?’. So that’s been really helpful for me.”* [10].

This collaboration could also confirm staff members’ existing good practices when engaging with victim-survivors. Reflecting on their communication with victim-survivors, one participant said: “I think it was quite reassuring that what I have been doing in the past, I’ve been on the right track.” [13].

Importantly, some participants felt that operating alongside the service better served the person experiencing the process:

*“It always helped having someone who was skilled in the area to provide that support independently of me because obviously from a legal perspective… it just didn’t probably feel as relatable as someone who might be able to assist on a more of an emotional*,* personal level. And that divide now that we have*,* I understand from other matters*,* [has] been very helpful in that respect.” (05)*.

#### Theme 2: changes to interactions with victim-survivors (notifiers)

The additional support for engagement work led to nearly all participants identifying changes to their interactions with victim-survivors as a result of engagement with the service.

Primarily, participants said that their experiences with the service had increased their awareness of, or reiterated the importance of, quality communication.

*“It’s made me think a lot more about how I’m going to communicate with that person. Whether I should call them*,* send them an email or be a bit more cognisant about how I deliver information and what sort of information I should give them.” (03)*.

Several participants talked about how their communication had become more intentional and had expanded to involve a deeper understanding of the victim-survivor’s circumstances. They reported using this knowledge to assist in making peoples’ experiences better “in a way that was positive and constructive for [them]” [13].

*“I probably give a little bit more thought to how I’m going to approach communication because those are the sorts of things that [social workers] will give a lot of thought to*,* and so if you’re planning communication or you’re planning a phone call or you’re planning an email jointly*,* they put so much thought into it that you automatically kind of take a little bit of that on.” (01)*.

#### Theme 3: improved perception of workload

Participants indicated that cases eligible for the support from the service typically involved a significant workload.

*“Wherever you have a sensitive matter with a notifier that requires support*,* or a practitioner that requires support*,* those cases will inevitably take up more of your time and energy because that is necessary.”* [13].

In these cases, participants described improved perception of workload due to service staff taking on specific aspects including additional communication or updates.

*“It assisted me greatly to have somebody else who could take some of that responsibility for engagement with vulnerable people off of my plate every now and then because I just don’t have time.” (02)*.

*“Really I see it as a little bit of weight off my shoulders*,* because the [service] is there to give them more support*,* give them more of an understanding about what the process looks like again because this is such an overwhelming process*,* so it takes a little bit of weight off me in that regard. So I would probably have to do less explaining*,* some less repetitive explaining.”* [10].

Importantly, support from the service had impacted the way in which communication was offered to victim-survivors, which also seemed to work better for staff members:

*“So before [the service] like we’d obviously say*,* ‘Oh you know*,* you can ring us whenever you’ve got any questions’ but now with the support service we’re able to book things in with people at a frequency that works for them. So it has really helped with that and also I feel like it’s probably a bit of workload off us as well.” (09)*.

This was particularly relevant for complicated cases, those that involve multiple victim-survivors, and those that involve a tribunal hearing where witnesses may have to give evidence.

*“I have a matter at the moment where I’ve got*,* I think it’s 10 notifiers and witnesses. So if [the] notifiers and witnesses do not qualify for the [support service] because they’re not - it’s not a sexual boundary violation*,* that takes up a huge chunk of time when it comes to me having to update them every month or every two months. There’s a lot of worries that they [notifiers] have around that*,* that I’m not now having to manage. I can give the information to the support service… they manage all of those communications. It’s a big help from my workload perspective”. (04)*

The presence of a dedicated support service allowed investigators in particular to focus more on their scope of work:

*“Sometimes they’ll [notifiers] throw really emotional kinds of questions*,* like*,* ‘Do you believe me?’ and as investigators*,* you know*,* we have to be really careful to be impartial. And it’s not about whether we believe you or not but [the service] are able to take on more of that caring sort of role*,* and explain that a lot better*,* so that the [investigator] can strictly just deal with the facts.” (06)*.

*“It adds to the*,* you know*,* emotional load that you’re carrying*,* dealing with these types of… files. They often want to become like your best friend. And so it can be hard I think sometimes for [staff] to keep that line clear and not blur it*,* which is where [the service] is great because they can do the*,* you know*,* ‘Let’s debrief how you’re actually feeling about it’ rather than the [staff]*,* which are like ‘I need to know what actually happened.” (06)*.

This delineation of role and scope was particularly evident in examples where a staff member and social worker made joint calls with victim-survivors. Staff were able to run through any updates required, or, for example, take a statement from the victim-survivor with the social worker present, then leave the call and the social worker to debrief with the person about the content they had just shared.

#### Theme 4: personal benefits of collaboration

Several personal benefits of working with the service were highlighted by participants. For some, seeing evidence of the effectiveness of service was satisfying:

*“I’ve been very grateful*,* particularly one example I know that she’s really grateful to have that ongoing contact from the [service]… they’re still in contact and updating them about the process. I guess it’s that continuity of contact… I think they seem to be quite appreciative of it.”* [12].

For others, collaborating reduced the stress associated with difficult cases or challenging communication:

*“And you worry about them*,* you know. Like you want to – you want to make sure they’re okay. So having the social workers as a point of contact takes away a lot of that. Like I found that quite stressful having to deal with them directly and not having any support available. The support service you know*,* they’re not counselling them or providing mental health treatment. But they know how to de-escalate situations and what to say.” (08)*.

Participants also benefitted from positive working relationships with service staff, and the informal collegial support provided by social workers.

*“They kind of just check in with us*,* that’s just something that they do as colleagues I guess*,* but they are professionals at that. So they will sort of check in with us and see how we’re going on matters or when we do have difficult witnesses or difficult matters*,* they’re a support to us as well even though that’s not part of their job.” (01)*.

### Negative case analysis

Some participants shared perspectives which differed greatly from the experiences of other staff interviewed. One participant who described limited engagement with the service on two cases felt that their existing skills and capabilities precluded the need for the service in some ways.

“*Because of my background […] I feel like I'm in a good position to be able to communicate with these people and put them at ease and build rapport.*”

*“If you’re decent at your – being able to communicate with people*,* I don’t know how much extra that they [the service] add.”* [11].

This participant expressed that victim-survivors often preferred engaging with them rather than being referred to additional support services, owing to previous rapport built. This participant, who had previously worked in settings with similar support services, opined that such services provide limited inputs. Their views contradicted others’ thoughts on the delineation of roles and the benefits that collaboration with the service could bring.

Further analysis highlighted examples of mixed sentiment in the data, or participant responses that did not cohere with some elements of the broader narrative. For example, one participant did not report any changes to the way they interacted with victim-survivors, though they did identify other benefits that had come from the service’s introduction:

*“There’s not really much of a difference with how I communicate or how often I communicate with witnesses who are in [the service] compared to those who aren’t in my current caseload.”* [10].

Like the more overt negative case, this participant felt that they had appropriate skills to provide support to people involved in the process, but distinguished that it was not necessarily their role:

*“I think ultimately that this process - investigations are so incredibly overwhelming*,* and I like to think that I’m quite hands on with the way that I build rapport and my explanations and updates with notifiers*,* but there is still a degree of separation*,* I think*,* because I am an investigator here*,* and I honestly don’t have the time or capacity to be giving them that extra support the [service] does.”* [10].

Additionally, one legal staff participant was reluctant to identify impacts from the introduction of the service but highlighted that it benefited those involved. This participant also described how experiences handling the work before the introduction of the service might have influenced staff perceptions:

*“I don’t know that you’re going to get any lawyers who have the insight into their psychology that would be able to provide you with that information because when you do this work for such a long time*,* it’s what we do*,* so random stuff rocks up and we deal with it… so I don’t know what the impact is necessarily. But what I can say is that I think it benefits everybody involved and I appreciate the additional resource and input.” (02)*.

The divergent perspective and mixed sentiment demonstrate that the benefits identified by the study were not universal. Whilst this divergence appeared largely to result from participants’ experiences of work with victim-survivors prior to Ahpra, it is an important contribution to understanding of the reception and implementation of the new service.

## Discussion

While there has been some research attention given to experiences of service users being supported through complex processes [25, 26], limited published literature is available about the experience of skilled staff members following the introduction of such a service. This is especially the case for sociolegal collaboration, where collaboration and outcomes are not well documented [9], as compared to health service environments. This makes transferring the findings of this research to wider settings challenging, however there remain takeaways about the introduction of trauma-informed services that are relevant to organisations and health systems more broadly.

This study found that the service was received positively overall and confirmed that staff saw a need for support in this space. Staff who had engaged with the service reported feeling supported in their case work, identified changes to the ways they communicated with others, and recognised personal and professional benefits.

Staff who engaged with the service identified benefits beyond support for victim-survivors, and reported feeling supported in their case work and communicating more thoughtfully with others. Such collaboration has positive implications for regulatory staff workflow and enhanced interactions with victim-survivors, as well as improved working relationships throughout the organisation.

The increased confidence and capability building identified by staff members in their approach to communication with victim-survivors involved in a regulatory process aligns with literature surrounding outcomes of trauma-informed interventions and training more broadly. For example, trauma-informed training was found to improve patient-centred interactions in a randomised trial with primary care providers in the United States [27]. A study with frontline health workers showed increases in confidence and reduced anxiety about working with trauma [28]. Similarly, the wellbeing benefits reported by participants in this study, including seeing the positive impact of the service, reduced stress and positive working relationships, are consistent with accounts of sociolegal collaboration helping to mitigate professionals’ vicarious or secondary trauma [14].

While the dominant narrative was positive, the negative and mixed cases identified some barriers to collaboration and were associated with less-favourable sentiments than those expressed by the broader participant group. Unlike those who saw benefit from the clear delineation of roles resulting from social workers’ taking responsibility for socioemotional support, these participants felt their skills were sufficient and did not see added value from the service.

Other studies report similar themes. Individual staff attributes are a central factor in the effective implementation of trauma-informed care, particularly individual resistance to change [3, 29]. For example, staff relational characteristics and personal histories were associated with attitudes towards trauma-informed care in a child welfare workforce [30]. A recent overview of barriers and facilitators to interprofessional collaboration in primary care found that a lack of clear roles and fears relating to professional identity were two of the four main barriers reported in published reviews [31]. More specifically, research on healthcare and social work collaboration has shown that challenges to integration include differing professional cultures and lack of understanding of the social work role among healthcare providers [8].

Addressing these challenges requires strong leadership, interprofessional education, and supportive policies that promote collaboration and resource sharing [8]. Tools to improve interprofessional communication and recognition of other professionals’ skills can also be key facilitators for collaboration [31]. Organisations considering the implementation of trauma-informed services or ways of working may benefit from anticipating workforce attitudes towards these changes and planning how to address them for smoother integration.

### Limitations

This study presents qualitative findings from a specific cohort and, while results are transferable to similar settings, should not be generalised more broadly.

While comparison of this study’s findings with existing literature assists in verifying the results presented, further research in this area would bolster findings. For example, the data was collected relatively early in the service’s history and so repeating collection could demonstrate changes in experience and attitudes towards the service over time. While demographic data was not reported here to uphold confidentiality, ensuring further data collection includes diverse perspectives would also benefit this area of research.

As this project is part of an internal evaluation, staff were interviewed by research team members who are members of the same organisation but operate in separate directorates. This potentially introduced social desirability bias or acquiescent responses from the cohort, highlighting that the reflexivity and transparency [24] of the research team was especially important. Researchers made efforts to mitigate pressure to provide ‘positive’ responses by ensuring no pre-existing relationships with participants, clearly and repeatedly confirming participant anonymity and confidentiality, and collecting data using a semi-structured interview schedule not reliant on binary question types [32].

## Conclusion

This study is the first to report outcomes for staff who engage with a trauma-informed support service introduced to support victim-survivors during a regulatory process. The findings confirm that genuine engagement with trauma-informed ways of working can benefit regulatory and legal services staff, though some barriers to smooth integration exist. In line with other literature, this work suggests that individual backgrounds and attitudes towards staff roles and responsibilities can affect and sometimes hinder collaboration. These factors should be taken into account to aid effective implementation.

## Supplementary Information

Additional file 1: Interview schedule,.doc., questions and prompts guiding interviews.

Additional file 2: Table of themes,.doc., provides description and exemplars of themes identified through analysis

Additional file 3: COREQ checklist, PDF, provides information relevant to reporting qualitative research

## Data Availability

Deidentified data analysed during the current study may be available from the corresponding author on reasonable request.

## Abbreviations

Ahpra: Australian Health Practitioner Regulation Agency

## Footnotes

1 Here, ‘victim-survivors’ describes those involved in a regulatory investigation of a potential sexual boundary violation as the victim of the alleged misconduct.
