# Supplementary material for: Benefits of workplace collaboration with a trauma-informed support service: a qualitative study
Source: BMC Health Serv Res. 2025 Oct 1;25:1272. doi: 10.1186/s12913-025-13398-x (PMC12487268; doi:10.1186/s12913-025-13398-x)
Supplement: Supplementary file 1 — Additional file 1: Interview schedule,.doc., questions and prompts guiding interviews. [file 12913_2025_13398_MOESM1_ESM.docx]

Appendix 1:

*Introduction and preamble not included*

**Section 1: Implementation and process**

How did you initially become aware of the Service?

Do you think awareness of the Service is sufficient in the area you work in? Why/why not?

Has your understanding of the Service changed over time/throughout your engagement?

- What’s changed, and why?
- Have you had access to any training, context, or background materials? How useful have these been?

Tell me about the process of referring a notifier or witness to the Service. How did you go about making a referral?

- How easy was that process?
- How did the notifier or witness respond to the referral?

How have you judged cases as appropriate for referral?

- Have there been matters where you regret not referring, or not referring sooner? Why?

From your experience, do you believe referrals have been timely for notifiers/witnesses?

- What do you see as barrier/s to timely referral?
- What would need to be in place to support timely referral?

**Section 2: Outcomes**

*For participants*

How effective do you think the Service has been in supporting participants?

- What do you see as barriers to engagement with the Service?

Can you give me an example of a situation where you’ve observed the impact of engaging with the Service on a participant, positive or negative?

*For staff*

How has engaging with the Service changed the way you interact with notifiers and/or witnesses?

- Increased or decreased contact frequency?
- Increased or decreased contact duration?
- Has this increased or decreased workload in these cases?
- Made contact easier/harder with participants? Why do you think that is?

How well has engagement with the Service integrated with your existing work?

Have you noticed any changes in the way you work or approach matters since engaging with the Service?

Any constructive criticism or negative feedback? (design, implementation, organizational, support)

- Have you experienced any difficulty engaging with the Service?
- Have you experienced any difficulty communicating with the Service following engagement?

What would you like to see change in the way we support people who make a sexual boundary notification?

*Closing remarks not included*
