# Supplementary material for: Benefits of workplace collaboration with a trauma-informed support service: a qualitative study
Source: BMC Health Serv Res. 2025 Oct 1;25:1272. doi: 10.1186/s12913-025-13398-x (PMC12487268; doi:10.1186/s12913-025-13398-x)
Supplement: Supplementary file 2 — Additional file 2: Table of themes,.doc., provides description and exemplars of themes identified through analysis [file 12913_2025_13398_MOESM2_ESM.docx]

Additional file 2. Table of themes

| Theme or *Subtheme* | Description | Exemplar |
| --- | --- | --- |
| Additional support for engagement work | Participant reports of feeling supported by the service in elements of their work with victim-survivors. Collates examples of how that support occurs, and by what mechanisms participants felt supported. | “I'm not a trained psychologist or social worker […] my role is not to give them psychological assistance or social work assistance. And I obviously want them to feel comfortable and I don't want them to be distressed et cetera but I'm not – I would fully put my hand up and say I'm probably not the best person to do that and that's better suited to the [service].” (02) |
| *Assistance approaching contact* | Included examples of service colleagues taking on some contact work with people involved in the process as well as supporting regulatory staff to prepare for contact with victim-survivors. This preparation helped to reduce perceived pressure around the frequency or duration of that contact. | “The [service] has really been taking the lead and doing the more regular updates, and I've sort of only been involved when they've thought that it's more appropriate to have a legal point of view. It's obviously taking if off of me a little bit that I don't have to have such regular contact with [victim-survivor] because I know that the [service] is supporting her and I obviously liaise with [the service] to provide updates … yeah, definitely helps in that respect.” (12) |
| *‘Not alone’ on case* | Support through feeling that another well-qualified staff member was contributing to the management of a case. | “I wasn't alone [on matter]. We both had context of the [victim survivor] and their manner, personality, concerns, distress, expectations. As opposed to a case without it, we have – I have numerous, and have had numerous challenging cases with challenging practitioners or notifiers. And I guess when you're seeking support of a team leader or a manager, they're not in the conversation [with victim survivor] with you. It's a little bit different. So, I guess it's just – just from that physical connection.” (13) |
| *Reassurance of good practice* | Participants felt reassured that their communication and approach to managing a case had been supported by someone with additional, specialist expertise. | “We all talk with the [service] to form a strategy about how we're going and what we're going to be saying before we have conversations as well. So that's just reassuring. I mean obviously I would do that before I'd call people if I had a tricky conversation coming up, but it's much easier if you can have that plan with someone who has expertise in the area.” (04) |
| Changes to interactions with victim-survivors (notifiers) | Participants speaking about making adjustments to their communication or interactions with victim-survivors. Included practical examples – reflecting that their communication had become more strategic, sensitive or structured – but also reiterating the importance of intentional communication. | “I obviously save the [correspondence] and I pull bits from it when I need to even respond. And I have just found that helpful particularly around the detail in explaining exactly what the process is and why we need their engagement without scaring them off with too much legal jargon. It definitely has helped me and [social worker’s] insights have assisted me in softening correspondence I suppose, I think that's the best way to describe it.” (05) |
| Personal benefits of collaboration | Benefits to staff resulting from engaging with the service. Wellbeing benefits were reported from positive working relationships as well as additional support from social workers as colleagues such as informal check ins with staff. These included intangible personal benefit derived from seeing evidence of the utility of support for victim-survivors referred to the service. | “Something that [social workers] will often also do with us is they kind of just check in with us, that's just something that they do as colleagues I guess, but they are professionals at that so they will sort of check in with us and see how we're going on matters, or you know when we do have difficult witnesses or difficult matters, they're a support to us as well even though that's not part of their job.” (01) |
| Improved perception of workload | Positive impacts on perception of workload inherent to matters, particularly around communication frequency and responding to needs or preferences articulated by victim-survivors. Examples included social workers taking on updates with victim-survivors and process support beyond what is strictly necessary for the bounds of the regulatory staff member’s role, such as debriefing. | “So before we'd obviously say, ‘oh you know, you can ring us whenever you've got any questions’ but now with the support service we're able to book things in with people at a frequency that works for them. I know some people want more regular updates whereas other people don't really want to hear from us unless it's coming up to the hearing or that sort of thing. So it has really helped with that and also I feel like it's probably a bit of workload off us as well because we don't have to try and provide that more emotional support to keep people engaged... there's someone else who can assist with that who is obviously a lot more qualified than we are.” (09) |
